# Effect of feed restriction and refeeding on body condition, digestive functionality and intestinal microbiota in rainbow trout (*Oncorhynchus mykiss*)

**DOI:** 10.1007/s10695-023-01170-z

**Published:** 2023-01-21

**Authors:** Maria Messina, Lucilla Iacumin, Giulia Pascon, Francesca Tulli, Emilio Tibaldi, Gloriana Cardinaletti

**Affiliations:** Department of Agricultural, Food, Environmental and Animal Science, University of Udine, Udine, Italy

**Keywords:** Fasting, Intestine physiology, Fish growth, Gut microbiota, Actinobacteria, Proteobacteria

## Abstract

The aim of the present work was to investigate the influence of fasting and refeeding on body condition, gut physiology and microbiota in reared *O. mykiss*. Ninety-six fish were randomly allotted among three groups subjected to different feeding plan: C (control, fed for 5 weeks); R (restricted ration over 3 weeks followed by 2 weeks feeding); F (fasted over 3 weeks followed by 2 weeks feeding) in a well’s fresh water flow-through rearing plan. Sampling occurred at 0, 1, 2, 4, 7, 14 days during the refeeding period. At day 0 and throughout the feeding period until day 14, the weight of the fish was significantly affected by the feeding restriction. Feed deprivation reduced significantly the viscerosomatic and hepatosomatic indexes. Brush border membrane enzymes’ specific activity was modulated by feeding regimes until day 7, to level in all experimental groups at day 14. At the end of the restricted/fasted period, the microbiota of the C group was made up of 70% of Actinobacteria, 24% of Proteobacteria, 4.2% of Firmicutes and < 1% of Bacteroides, while the restricted and fasted group were characterized by a strong reduction of Actinobacteria, and a significant increase in Bacteroidetes and Firmicutes. The feed deprivation determined a dysbiosis, allowing the development of different commensal or pathogenic bacteria. In conclusion, the effects of 2 weeks of feed deprivation, excluding those related to body weight, are gradually mitigated by refeeding, which allows the restoration of digestive functions and a healthy intestinal microbiota.

**Supplementary Information:**

The online version contains supplementary material available at 10.1007/s10695-023-01170-z.

## Introduction

Wild fish experience periods of starvation or feed restriction for several reasons such as the physiological state related to the reproductive season (prespawning stage, spawning migration), and adverse environmental conditions (winter months) (McCue 2010). Reared fish suffer a fasting period for depuration to reduce handling stress before transport and slaughtering or in case of poor water quality (Davis and Gaylord 2011), in overproduction conditions and even to prevent mortality caused by disease outbreaks (Shoemaker et al. 2003). During starvation or fasting, an imbalance between energy expenditure and energy input occurs, especially over a long period, when the animals must mobilize their energy stores. Compensation strategies for a condition of low energy and protein supply at the resumption of feeding are numerous. Fish can use hyperphagia (Ali et al. 2003; Sevgili et al. 2012, 2013) sustained by a different endocrine status, thanks to an increase in orexigenic signals (Won and Borski 2013). This strategy has been reported to improve feed utilization in channel catfish and sea bream (Gaylord and Gatlin III 2001; Bavčević et al. 2010). On the other hand, an improvement in feed conversion efficiency has not been registered in artic charr, hybrid sunfish, hybrid tilapia and gibel carp after a period of hyperphagia (Miglav and Jobling 1989; Hayward et al. 2000; Wang et al. 2000; Xie et al. 2001). Moreover, Boujard et al. (2000) reported high feed efficiency without hyperphagia during the refeeding period in rainbow trout and Peres et al. (2011) observed nor overfeeding nor differences in feed efficiency ratio in sea bream.

As other vertebrates, fasted and refed fish can experience a process of compensatory growth (CG) with accelerated somatic growth (Ali et al. 2003) regardless of hyperphagia. CG is a well-known phenomenon in salmonid fish (Johansen et al. 2001; Nikki et al. 2004; Krogdahl and Bakke-McKellep 2005; Sevgili et al. 2012, 2013; Tasbozan et al. 2016; Cassidy et al. 2018), but limited information is available on gut physiology (Krogdahl and Bakke-McKellep 2005) and intestinal microbiota of fish during the refeeding period (Sakyi et al. 2021). These aspects are of utmost importance when modulation of feeding plans is applied under controlled rearing conditions in order to improve the environmental, economic and managerial sustainability of fish farming.

The enzymes of the intestinal brush border membrane (BBM) have already been considered in order to study the relationship between gut physiology and feeding habits (Harpaz and Uni 1999), the effects of experimental diets in different reared fish species (Harpaz et al. 2005a, b; Tibaldi et al. 2006; Messina et al. 2019) and the effect of feed deprivation in sea bass (Hakim et al. 2009). They play a key role in the final stages of digestion, as the disaccharases maltase-glucoamylase (MALT) and sucrase-isomaltase (SI) produce glucose and fructose, γ-glutamil transaminase (γ-GT) is involved in the amino acid metabolism, and intestinal alkaline phosphatase (IAP) is an indicator of enterocyte development (Gisbert et al. 2018) and is responsible for the dephosphorylation of nutritional compounds (Villanueva et al. 1997).

In the last decade, the study of gut physiology has also taken into account the intestinal microbiota and the relationship between host and microbial community in mammals (Gaboriau‐Routhiau and Cerf‐Bensussan 2016; Chung et al. 2012) and in fish (Gajardo et al. 2017; Bruce et al. 2018; Parshukov et al. 2019; Wang et al. 2020). Microorganisms support the animals to digest fibres, supply vitamins, trigger the intestinal immune system and resist the attacks of the pathogens thus maintaining a healthy gut environment. To our knowledge, the behaviour of the intestinal microorganisms during a period of fasting and refeeding still needs to be studied in reared rainbow trout.

In this context, the current study aimed to examine how rainbow trout (*Oncorynchus mykiss*) faces the resumption of feeding after a period of feed restriction with a focus on the fish welfare and gut condition. To this end morphometric parameters, plasma metabolites, the activity of intestinal BBM enzymes and the composition of the gut microbiota were investigated.

## Materials and methods

### Ethical statement

The animal study was in strict accordance with the recommendations of the European Guidelines 2010/63/EU on the protection of animals used for scientific purposes. All animal handling procedures were approved by the Ethics and Animal Care Committee of the University of Udine, permit number 2/2019.

### Experimental design and sampling procedures

Trout, purchased from a commercial farm (Azienda Agricola Salvador, Fontanafredda (PN), Italy), were reared at the facility of the department of Agricultural, Food, Environmental and Animal Science of the University of Udine. After 2 weeks’ acclimation, 96 trouts (initial body weight 129.1 ± 3.5 g) were randomly distributed among 6 tanks (16 fish/tank) each supplied with 8 L min^−1^ of well water in a flow-through system.

Fish were fed a commercial trout feed (crude protein 44.0%, crude fat 22.0%, crude fiber 2.7%, Excel, Skretting Italia, SpA; Table S1) and were subjected to different feeding plan: C, fed over 5 weeks (1.3% body weight); R, restricted ration (30% of C ration, corresponding to 0.4% body weight and under the maintenance needs) over 3 weeks followed by 2 weeks feeding at 120% of C ration; F, fasting over 3 weeks followed by 2 weeks feeding at 120% of C ration. A fixed ration during the refeeding period was applied to maintained fish at the same condition. The value of 120% (corresponding to 1.56% of the initial body weight) follows previous observations made in the same plant and in the same environmental conditions aimed at verifying what ration the fish began to refuse pellets.

Two tanks were randomly assigned to each treatment/feeding plan. Fish were carefully hand fed according to the selected ration once a day, at 9.30 am, 6 days a week.

Water temperature, measured on a daily basis, was constant throughout the experimental period (12.7 °C). The constancy of this parameter is a key factor in such experiments, because in fish it determines the metabolic rate and the consequent energy demand, with significant effects on loss in the body mass especially in fasting fish (McCue 2010). During the trial water quality parameters (dissolved oxygen 9.3 ± 0.57 mg/L, pH 7.97 ± 0.35, total ammonia nitrogen < 0.08 mg/L, nitrite-nitrogen < 0.015 mg/L) were monitored on a weekly basis and a constant photoperiod of 12 L:12D was applied with artificial fluorescent light at 400 lx.

At the end of the 3-week restriction/fasting period (T0) and on day 1, 2, 4, 7 and 14 of refeeding (T1, T2, T3, T4, T7, T14), 24 h after the last meal, two fish per tank (four fish per treatment) were netted and euthanized, with a lethal dose of MS-222 (300 mg/l), and blood samples were immediately withdrawn from caudal vessels into heparinized tubes. Plasma, obtained by centrifugation at 1500 × g at 4 °C for 15 min, was stored at − 20 °C until the analysis.

After blood sampling, fish were measured for body weight and total length to calculate the condition factor (K, weight/length^**3**^ × 100). Afterwards, the digestive tract was removed from the open abdomen and viscera and liver were weighed to calculate viscerosomatic index (VSI, viscera weight/body weight × 100) and hepatosomatic index (HSI, liver weight/body weight × 100). The digestive tract was isolated and digesta were gently squeezed out from the intestine, which was divided into pyloric caeca (PC), proximal intestine (PI, section below the tract with PC until the increase in diameter indicating the start of the distal intestine) and distal intestine (DI, the terminal part of the intestine with larger diameter, till the anus), rinsed with iced saline, gently dried with a piece of paper and stored at − 20 °C until the analysis.

### Plasma metabolic parameters

The plasma metabolites glucose (Glu, mg × dL^−1^), cholesterol (Chol, mg × dL^−1^), triglycerides (Trig, mg × dL^−1^), total proteins (TP, g × dL^−1^) and albumin (Alb, g × dL^−1^) were determined by an automated analyser system for blood biochemistry (Roche Cobas Mira, Biosys, Milan, Italy) and commercially available kits (Biochemical Enterprise, Milan, Italy), following the manufacturer’s protocols.

### Specific activity of BBM enzymes

The extraction of the BBM enzymes from the three sections of the gut and the activity of the enzymes MALT, SI, γ-GT e IAP was determined according to Messina et al. (2019). One unit of enzyme activity is the amount of enzyme that transforms or hydrolyses 1 µmole of substrate ml^−1^ min^−1^. Specific enzyme activity, U, was calculated as enzyme activity × mg^−1^ of protein.

### Gut microbiota profile

Following the samples procedures explained above, part of the proximal intestine was dissected under aseptic conditions at T0, T7 and T14. The intestine was softly emptied and the content (faeces and mucous) immediately extracted in triplicate. Time between euthanasia and intestine content extraction was less than 5 min. Microbial DNA from gut content samples was extracted using the Fecal DNA MiniPrep kit (Zymo Research; Irvine, CA, USA), following the manufacturer’s instruction. DNA concentration was measured using a NanoDrop ND-1000 spectrophotometer (Thermo Fisher Scientific, Milan, Italy) and standardized to 100 ng/μL. The replicates per each time were pooled in order to be processed by NGS. Partial 16S rRNA gene sequences (V3 region) were amplified from extracted DNA using primer pair Probio_Uni and/Probio_Rev, according to the protocol developed by Milani et al. (2013). Shortly, after amplification the integrity of the PCR amplicons was analyzed by electrophoresis on an Experion workstation (BioRad, Milan, Italy). Purification of the amplicons was performed by electrophoretic separation on a 1.5% agarose gel and the use of a Wizard SV Gen PCR Clean-Up System (Promega, Milan, Italy). In order to remove primer dimers, a further purification step involving the Agencourt AMPure XP DNA purification beads (Beckman Coulter Genomics GmbH, Bernried, Germany) was applied. Sequencing of the 16S rRNA gene was performed using a MiSeq (Illumina, GenProbio srl, www.genprobio.com) (Milani et al. 2013). The obtained fastq files were processed using a custom script based on the QIIME software suite (Caporaso et al. 2010). Paired-end read pairs were assembled to reconstruct the complete Probio_Uni/Probio_Rev amplicons. Quality control retained sequences with a length between 140 and 400 bp and mean sequence quality score > 20, while sequences with homopolymers > 7 bp and mismatched primers were omitted.

The bacterial profile at phylum, family and genus level was reported as relative abundance. Moreover, the bacterial profile at species level was predicted, which is to be considered as approximate.

In order to calculate downstream biodiversity measure (alpha diversity index), 16S rRNA Operational Taxonomic Units (OTUs) were defined at 100% sequence homology using DADA2 (Callahan et al. 2016); OTUs not encompassing at least 2 sequences of the same sample were removed. Notably, this approach allows highly distinctive taxonomic classification at single nucleotide accuracy (Callahan et al. 2016). All reads were classified to the lowest possible taxonomic rank using QIIME2 (Caporaso et al. 2010; Bokulich et al. 2018) and a reference dataset from the SILVA database (Quast et al. 2013). Chao1 and Shannon indexes, as well as Observed OTUs and Good’s coverage, were calculated for 4 sub-samplings of sequenced read pools and represented by rarefaction curves. Beta diversity was evaluated with the phylogeny-based UniFrac distance metric (Lozupone and Knight 2005) and visualized using Principal Coordinate Analysis (PCoA) plots.

### Statistical analysis

Data on body weight, K, VSI and HSI, plasma metabolites and specific enzymatic activities were subjected to one-way ANOVA with treatment as a fixed factor and, if necessary, means were compared using the Duncan’s test, (significant level 95%). All analyses were completed using the SPSS package (SPSS Inc., Chicago, IL, USA). Overall data on intestinal microbiota were analysed by R vers. 4.0.4, pheatmap (Kolde 2019) package using Pearson correlation as distance metric to perform hierarchical clustering analysis.

## Results

### Growth performance

No mortality was recorded during the experiment. At the end of the 3 weeks of treatment (T0) the groups R and F had lost about 24 and 44% of body weight respectively, resulting in both significantly different from group C (Table 1). Refeeding trout for 2 weeks at 120% of the C ration resulted in a partial compensation of body mass at the different sampling times. Nevertheless, fish of the R and F group still exhibited significantly lower body weight compared to C group, after 14 days (− 11% and − 18%, respectively).

**Table 1:** Growth parameters and morphometric indices of trout at the end of the restriction/deprivation period (T0) and at the subsequent sampling times (T1, T2, T4, T7, T14) (*n* = 4).  Data are presented as mean ± SD

Parameter	Treatment	T0	T1	T2	T4	T7	T14
Weight g	C	189.5^a^ ± 27.25	199.0^a^ ± 8.06	205.9^a^ ± 24.12	220.6^a^ ± 31.17	225.3^a^ ± 21.06	231.1^a^ ± 14.02
	R	143.8^b^ ± 7.01	152.0^b^ ± 7.03	159.1^b^ ± 21.89	172.1^b^ ± 3.01	171.8^b^ ± 9.89	206.1^b^ ± 1.72
	F	125.2^b^ ± 15.86	127.4^c^ ± 12.81	144.1^b^ ± 16.64	149.2^c^ ± 2.51	153.4^b^ ± 19.79	190.3^b^ ± 7.36
	P	0.001	0.000	0.006	0.000	0.002	0.005
K	C	1.13 ± 0.02	1.19 ± 0.07	1.21^a^ ± 0.06	1.17 ± 0.04	1.23 ± 0.12	1.21 ± 0.30
	R	1.05 ± 0.04	1.20 ± 0.13	1.09^b^ ± 0.05	1.17 ± 0.03	1.19 ± 0.11	1.23 ± 0.05
	F	0.97 ± 0.05	1.05 ± 0.09	1.05^b^ ± 0.01	1.14 ± 0.06	1.19 ± 0.06	1.23 ± 0.12
	P	> 0.05	> 0.05	0.011	> 0.05	> 0.05	> 0.05
VSI	C	13.42^a^ ± 0.51	13.54 ± 1.37	12.70^b^ ± 1.77	13.05 ± 0.95	12.60^b^ ± 2.00	11.74^b^ ± 0.75
	R	10.44^a^ ± 1.02	14.26 ± 2.81	15.37^a^ ± 0.62	15.99 ± 1.40	13.65^b^ ± 2.10	14.69^a^ ± 0.72
	F	7.90^b^ ± 0.50	10.13 ± 0.59	15.47^a^ ± 0.67	15.46 ± 3.99	17.42^a^ ± 1.92	15.17^a^ ± 1.92
	P	0.023	> 0.05	0.042	> 0.05	0.035	> 0.05
HSI	C	1.26^a^ ± 0.11	1.35^a^ ± 0.06	1.18^a^ ± 0.06	1.34 ± 0.20	1.15 ± 0.01	1.05^c^ ± 0.05
	R	1.16^a^ ± 0.05	1.36^a^ ± 0.18	1.14^a^ ± 0.18	1.30 ± 0.13	1.20 ± 0.05	1.39^b^ ± 0.08
	F	0.76^b^ ± 0.21	0.82^b^ ± 0.05	0.85^b^ ± 0.10	1.23 ± 0.08	1.28 ± 0.27	1.61^a^ ± 0.17
	P	0.022	0.004	0.029	> 0.05	> 0.05	0.002

Column means with different letters are significantly different (a, b, c, *P* < 0.05)

*C*, control; *R*, restricted; *F*, fasted; *K*, condition factor; *VSI*, viscerosomatic index; *HSI*, hepatosomatic index

The K values were unresponsive both to the dietary restrictions and to the refeeding with the exception on T2 when the condition factor resulted lower in R and F groups than in the C group (Table 1).

Three weeks of feed deprivation reduced significantly the viscerosomatic and hepatosomatic indexes in the F group (Table 1, T0). On day 7 and 14 of refeeding the VSI in the F group was significantly higher than in the C group (*p* < 0.05), while in group R the value of VSI exceed that of group C at T2 and T14. The HSI in the F group was significantly lower compared to the C and R groups (*p* < 0.05) after 1 and 2 days refeeding, and reached similar values to C and R groups at day 4 and 7. The highest value of HSI was reached by the F group, after day 14 of refeeding. The R group showed the same pattern of the C group until day 14 when the HSI value was significantly different from both C and F groups (1.05 < 1.39 < 1.61 for C, R and F group respectively; *p* < 0.05). It is interesting to notice that as long as the refeeding days increased viscera and liver weight assumed higher values in R and F groups than in C group, resulting in significantly higher VSI and HSI (Table 1).

### Plasma metabolites

Plasma metabolites showed different patterns during the 2 weeks of sampling times (Table 2). Trout were able to maintain the level of glucose during both the feed-deprivation and the refeeding period. On the contrary, 3 weeks of reduction/fasting (T0) affected circulating triglycerides concentration with the lowest values in R and F groups (*P* < 0.05). During the refeeding period restricted and fasted fish were able to restore triglyceride plasma concentration starting from T1, after the first meal at 120% of the C ration. Plasma cholesterol in F group was affected by the treatment at the end of the fasting period (T0) and throughout the sampling time until T7, with significantly lower values than the C group.

**Table 2:** Plasma metabolic parameters of the experimental groups at the end of the restriction/deprivation period (T0) and at the subsequent sampling times (T1, T2, T4, T7, T14) (n = 4). Data are presented as mean ± SD

Parameter	Groups	T0			T1			T2			T4			T7			T14		
Glu mg/dL^−1^	C	102.0	±	13.3	92.0	±	9.5	86.3	±	5.8	97.0	±	11.7	92.7	±	2.9	92.3	±	3.5
	R	77.7	±	27.2	93.3	±	11.2	75.0	±	5.2	88.0	±	13.1	86.0	±	9.8	92.7	±	13.6
	F	71.3	±	5.5	105.7	±	16.5	87.3	±	21.7	83.0	±	15.1	78.3	±	2.1	96.3	±	13.1
	P	> 0.05			> 0.05			> 0.05			> 0.05			> 0.05			> 0.05		
Chol mg/dL^−1^	C	258.0^a^	±	9.0	254.7^a^	±	7.6	226.3^a^	±	19.0	271.3^a^	±	33.3	212.3^a^	±	8.1	249.7	±	7.6
	R	203.3^a^	±	58.2	223.7^a^	±	30.9	191.0^ab^	±	20.4	214.3^b^	±	16.7	213.3^a^	±	19.7	249.7	±	33.3
	F	183.3^b^	±	18.7	171.0^b^	±	14.5	169.3^b^	±	23.7	178.7^b^	±	18.6	158.0^b^	±	8.7	199.0	±	17.1
	P	0.035			0.006			0.043			0.009			0.003			> 0.05		
Trig mg/dL^−1^	C	221.0^a^	±	26.63	273.3	±	37.65	227.7	±	104.8	284.7	±	14.98	249.3	±	22.5	236.3	±	9.5
	R	89.6^c^	±	7.06	305.3	±	49.08	367.7	±	135.41	327.3	±	61.13	321.0	±	10.54	294.3	±	42.90
	F	159.7^b^	±	42.77	272.7	±	88.10	283.3	±	78.93	451.3	±	96.34	270.3	±	30.53	280.7	±	16.44
	P	0.004			> 0.05			> 0.05			> 0.05			> 0.05			> 0.05		
TP g/dL^−1^	C	2.4	±	0.00	2.5	±	0.15	2.5	±	0.23	2.5	±	0.10	2.7	±	0.20	3.0	±	0.10
	R	2.1	±	0.36	2.3	±	0.10	2.3	±	0.35	2.2	±	0.15	2.7	±	0.06	2.9	±	0.17
	F	2.3	±	0.32	2.2	±	0.12	2.4	±	0.17	2.5	±	0.10	2.4	±	0.06	3.0	±	0.00
	P	> 0.05			> 0.05			> 0.05			> 0.05			> 0.05			> 0.05		
Alb g/dL^−1^	C	1.8	±	0.12	1.8	±	0.15	1.4	±	0.12	1.5	±	0.12	1.7	±	0.10	1.7	±	0.00
	R	1.4	±	0.20	1.7	±	0.06	1.3	±	0.31	1.4	±	0.10	1.6	±	0.00	1.8	±	0.15
	F	1.4	±	0.15	1.5	±	0.12	1.4	±	0.06	1.5	±	0.12	1.5	±	0.06	1.9	±	0.06
	P	> 0.05			> 0.05			> 0.05			> 0.05			> 0.05			> 0.05		

Column means with different letters are significantly different (a, b, c, *P* < 0.05)

*C*, control; *R*, restricted; *F*, fasted

Otherwise, plasma cholesterol in the R group showed no changes during feed reduction and refeeding, except on T4. Circulating cholesterol had similar concentrations in the 3 groups, after 14 days at 120% of the C ration. In relation to plasma protein and albumin no conclusive changes were found throughout the experimental period.

### Specific activity of BBM enzymes

**Fig. 1:**
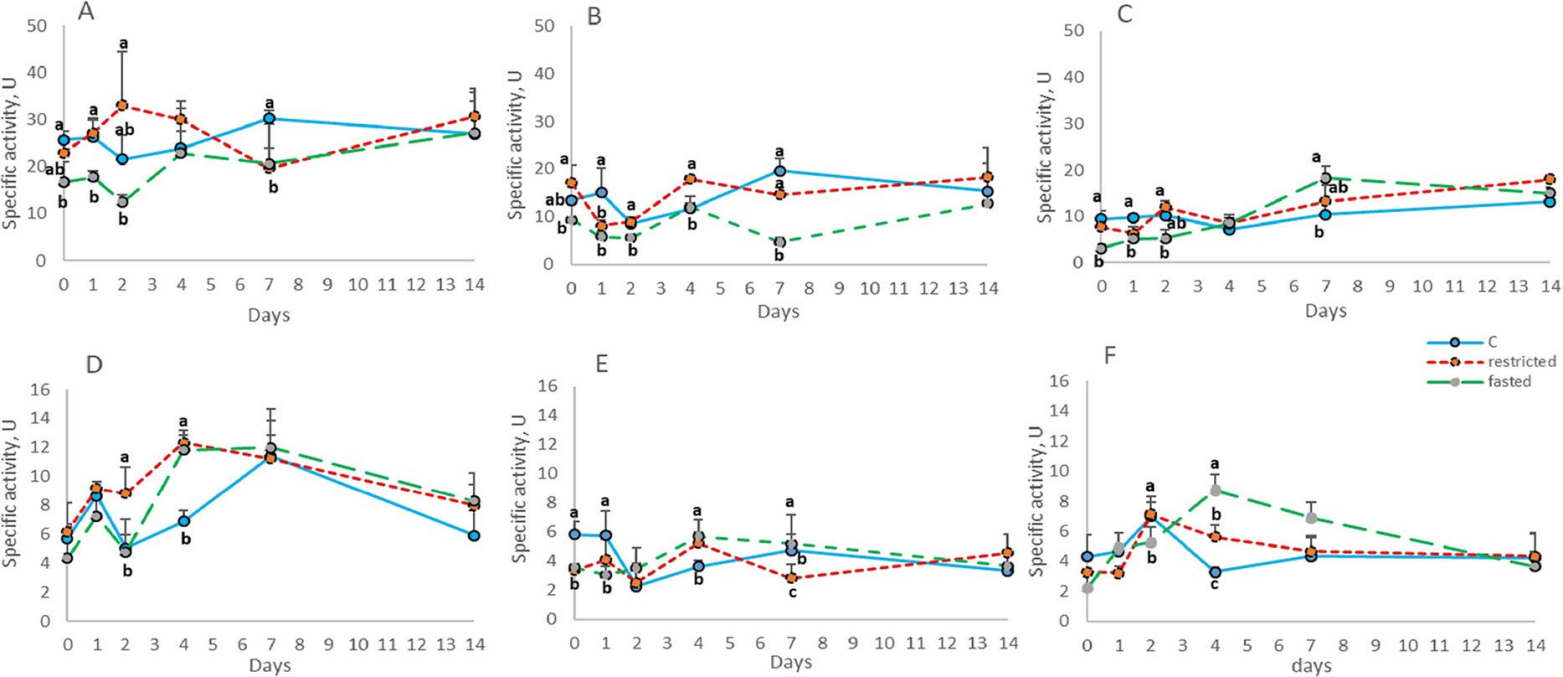
Specific activity of maltase in pyloric caeca (A), anterior intestine (B), posterior intestine (C), and sucrase-isomaltase in pyloric caeca (D), anterior intestine (E) and posterior intestine (F) of the experimental groups during the refeeding period. Data are presented as mean + SD (*n* = 4). Different letters indicate significant differences among experimental groups (*p* < 0.05)

The analysis of the BBM enzyme activities in all the intestinal tracts showed significant variations (Fig. 1). The average specific activity of maltase-glucoamylase, regardless the treatments, was higher in the PC than in the PI and the DI (24.20 ± 5.41U, 12.20 ± 4.61U and 10.10 ± 4.26 U, respectively). Overall, the activity of MALT in the three tracts of the intestine was significantly lower in the previously fasted group than in the C group. The pattern of the dietary restricted fish presented similar values compared to the C group, with the exception of T7 in the PC (Fig. 1A). The activity of this enzyme was restored in all the treatments and tracts on day 14.

The average specific activity of sucrase–isomaltase, regardless the treatments, showed a similar trend to that of MALT, with the higher value in the PC than in the PI and DI (8.22 ± 2.66 U, 4.05 ± 1.14 U, and 4.87 ± 1.67, respectively). In general, the activity of sucrase-isomaltase of both restricted/fasted groups shared similar patterns compared with the C group. This enzyme showed similar values in all the treatments and tracts on day 14 (Fig. 1D, E, F).

**Fig. 2:**
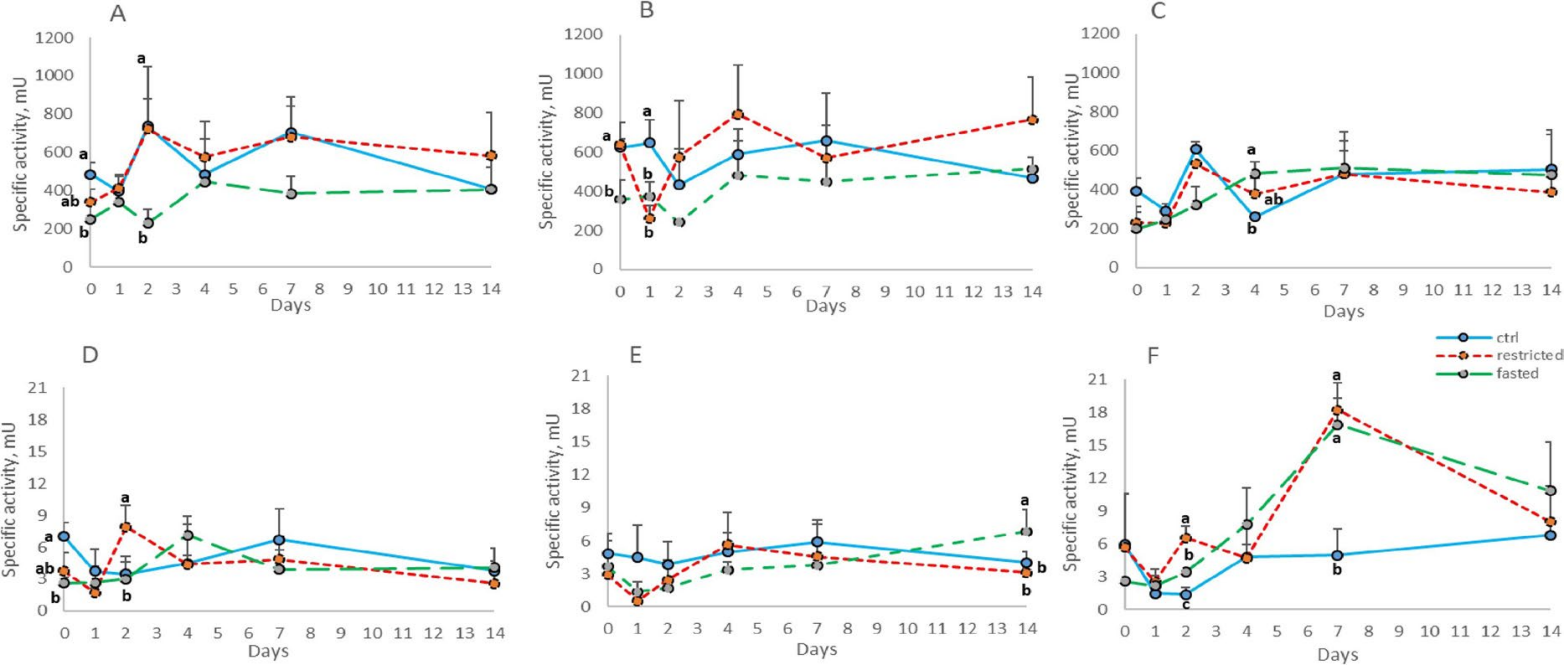
Specific activity of intestinal alkaline phosphatase pyloric caeca (A), anterior intestine (B), posterior intestine (C), and γ-glutamil transaminase in pyloric caeca (D), anterior intestine (E) and posterior intestine (F) of the experimental groups during the refeeding period. Data are presented as mean  + SD (n = 4). Different letters indicate significant differences among experimental groups (*p* < 0.05)

The feed deprivation period significantly affected the activity of IAP in the PC and PI (Fig. 2A, 2B, T0). In these two tracts all groups presented similar activity from day 4 of refeeding. A different pattern of IAP activity could be noticed in the DI (Fig. 2C) where the activity of the F group was significatively higher on T4.

The specific activity of γ-GT was limited affected by the feeding scheduled (T0) showing significant differences only in the PC (Fig. 2D). In this tract, except for a significant peak in the activity of the fasted group on day 2, the refeeding did not affect all the groups over 14 days. In the PI a similar trend can be observed in group R and F, although not different from C group, until day 14 when the highest activity of γ-GT was observed in the fasted/refed group. Conversely, in the DI the refeeding triggered a significantly higher activity on T2 and T7 in the R and F groups than in the C group. Finally, the activity of γ-GT reached similar values among the groups on T14.

### Gut microbiota profile

**Fig. 3:**
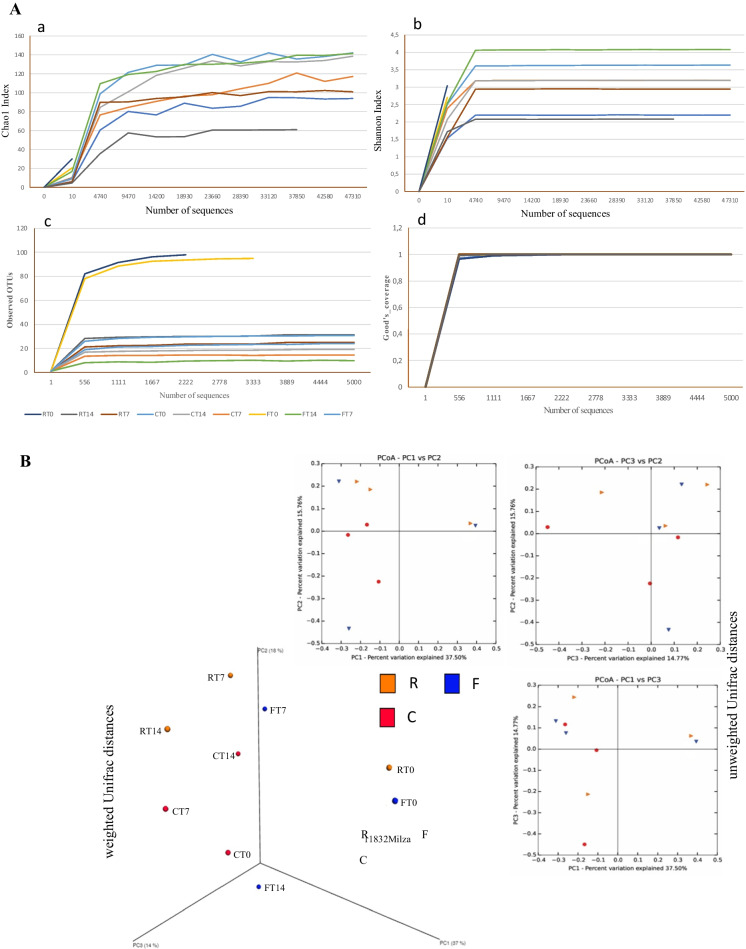
**Panel A**, Alpha diversity: Rarefaction curves, observed OTUs, Good’s coverage generated for 16S rRNA gene sequences obtained from the samples. (a) Represents the rarefaction curves using the Chao1 index; (b) displays rarefaction curves using the Shannon index; (c) observed OTUs (max depth of 5000 seq/sample); (d) Good’s coverage (max depth of 5000 seq/sample). **Panel B**, Beta diversity: Principal Coordinate Analysis (PCoA). Percentages shown along the axes represent the proportion of dissimilarities captured by the axes

Sequencing of the samples generated a total of 405,743 reads after quality filtering, with an average of 45,088 (range, 3220 to 88,348) sequences per sample and a median length of 180 (Accession number SRA data PRJNA815627). Alpha (within-sample) diversity was calculated at a maximum depth of 50,000 sequences per sample, with the rarefaction curves (Chao1, Shannon index, Observed OTUs e Good’s coverage) shown in Fig. 3 panel A. Considering that rarefaction curves reached a plateau, only few new sequences will be detected with increasing sequencing depth (a, Chao1 index and b, Shannon index). Therefore, it can be concluded that the entire microbial community was sufficiently covered in all the samples. In both curves, high values of the statistical index indicated a high complexity of the samples, regardless of the treatment and the sampling time. Since for samples FT0 and RT0 the curves plateau is not properly visible, Alpha diversity was also calculated at a maximum depth of 5000 sequences per sample, and this allowed to give clear evidence of the reached plateau for all the rarefaction curves.

Shannon diversity index represents how diverse the species in a given community are. It rises with the number of species and the evenness of their abundance and looking the curves differences among the samples can be observed. The Observed OTUs (count of unique OTUs in each sample) curves showed that the diversity observed with Shannon index could be attributed in particular at an increment of species diversity in samples FT0 and RT0 demonstrating that the diet directly affected species diversity and their abundance. Lastly, Good’s coverage index, used to estimate the percentage of total bacterial OTUs represented in a sample, being really close to 1 indicated that the depth of sequencing is enough for all the samples.

Principal coordinates analysis was used on weighted and unweighted Unifrac distances to examine beta diversity according to the clustering of samples (Fig. 3, panel B). Weighted Unifrac distances took into account abundance of each taxon, while unweighted distances are based only on presence/absence data (Lozupone and Knight 2005). RT0 and FT0 resulted different from the other samples, clustering in the same region of the space, demonstrating to be the most diverse in terms of both abundances of each taxon and presence/absence of taxon (*P* < 0.05, analysis of similarity). Vice versa, samples collected at T7 and T14, independently from the feeding regime, resulted similar to the control samples.

**Fig. 4:**
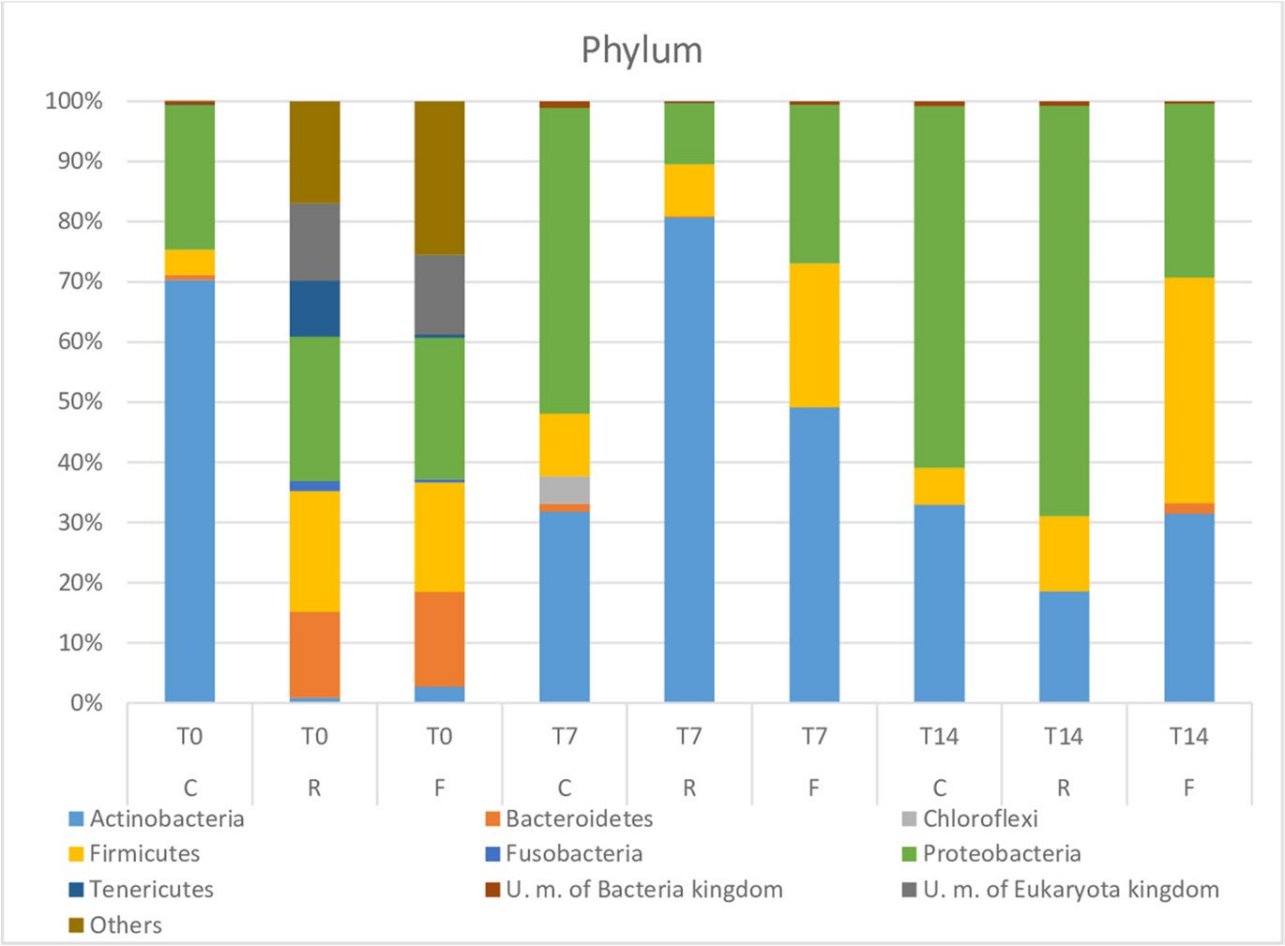
Relative abundance (%) of the intestinal microbiota of the experimental groups at the end of the restriction/deprivation period (T0) and at the subsequent sampling times (T7 and T14). Phylum level. C = Control, R = Restricted, F = Fasted

**Fig. 5:**
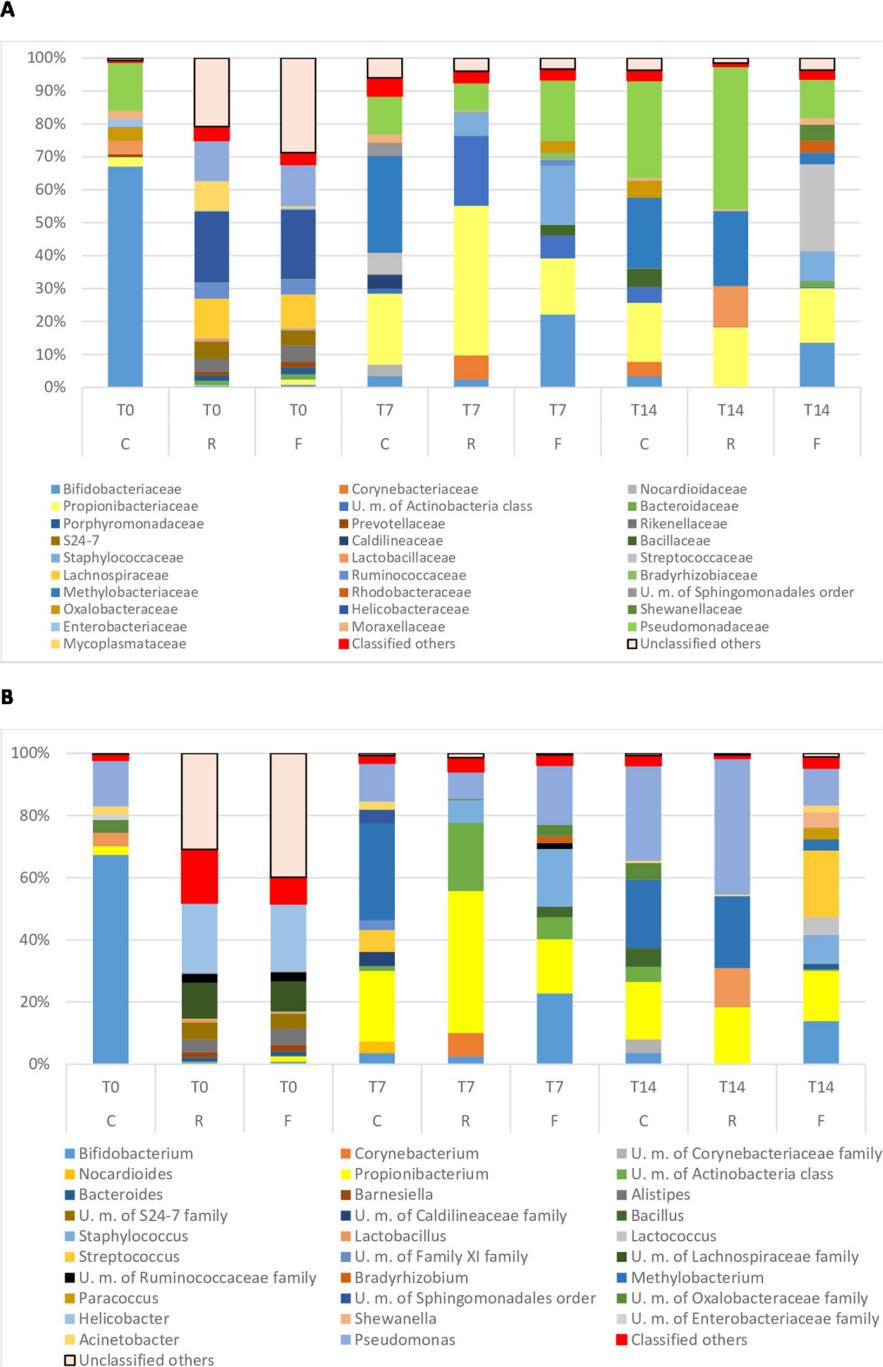
Relative abundance (%) of the intestinal microbiota of the experimental groups at the end of the restriction/deprivation period (T0) and at the subsequent sampling times, (T7 and T14). A = Family level. B = Genera level. C = Control, R = Restricted, F = Fasted

Looking at the taxonomy results (Figs. 4, 5), it is possible to evaluate if the differences among samples depend on variations in the relative abundance of genera/species or on the introduction of new microbes.

A common genus, which was always identified in all the groups, was *Pseudomonas* spp. Its percentage ranged from 14.86% on day 0 to 30.64% on day 14 in group C. Moreover, although present at levels of 0.11% and 0.4% on day 0 in groups R and F, respectively, *Pseudomonas* spp. reached the highest percentage of 43.86% in group R (T7) and the percentage of 19.14% and 12.10% in group F, on T7 and T14, respectively.

At T0, at the end of the restricted/fasted period for the R and F groups, the relative diversity and abundance of bacterial phyla in the anterior intestine were not similar between C and the treated groups R and F. The microbiota of the C group was made up of 70% of Actinobacteria, 24% of Proteobacteria, 4.2% Firmicutes and < 1% of Bacteroides and other microbial phyla, while R and F groups were characterized by a completely different population (Fig. 4). Actinobacteria phylum was reduced to 0.89% and to 2.73% in restricted and fasted group, respectively (T0). In both theses, Bacteroidetes and Firmicutes increased significantly, and, together with *Proteobacteria*, they represented the 60% of the population. The remaining 40% was represented by other unknown phyla and cells from Eukariota kingdom.

The situation changed after 7 and 14 days of refeeding. In fact, Actinobacteria succeeded in developing and regaining the lost space in R and F groups and, together with Firmicutes and Proteobacteria, they restored the microbial balance to a situation very similar to that of the control group, with some quantitative differences (C, T7).

Focusing on family and genera level (Fig. 5, A and B), the effect of the restriction/fasting period on relative diversity was more evident than when considering the phylum level. Bifidobacteriaceae (Fig. 5A), and *Bifidobacterium* spp. (Fig. 5B) in particular, represented 67% in C (T0) but were less than 1% in groups R and F at the same time. Anyway, it should be noted that in the control group, on days 7 and 14 this genus still dropped to values < 4%. In group R at T7, *Bifidobacterium* spp. remained lower than 3%, being even absent on day 14. Conversely, in group F *Bifidobacterium* spp. rose to concentrations of around 22%, and then dropped back to 14% at T7 and T14, respectively.

Other Lactic Acid Bacteria (LAB) were found and showed a variable distribution in the different thesis. The genus *Lactobacillus* was present at 4.19% on day 0 in the control group (Fig. 5, B and C, T0), and in group R on day 14 (Fig. 5B; R, T14), whereas in F it was found once at 0.5% on day 0 (Fig. 5B; F, T0). The genus *Streptococcus* was detected twice, in the control group (6.96%, T7) and in the fasted group (21.36%, T14), while *Lactococcus* was only present in the fasted group (5.83%, T14). The genus *Staphylococcus* was also found on day 7 in the R and F group (7.25% and 18.56%, respectively) and on day 14 decreased to 9.27% in the F group and disappeared in the R group (Fig. 5B; T7, R and F, and T14, F). It is worth noting that in the control group, this genus was never identified. Similarly, unknown members of Lachnospiraceae family were discovered on day 0 in group R and F, showing percentages of 11.70 and 9.73%, respectively, but in the control group they were never spotted (Fig. 5B; T0, R and F).

The genus *Methylobacterium* appeared on day 7 and 14 in group C (31.05% and 22.08%, respectively, Fig. 5B; C, T7 and T14), as well as on day 14 in both groups R and F (23% and 3.57%, respectively, Fig. 5B; R, T14 and F, T14). Among others, surprisingly the genus *Helicobacter* was also present. It appeared on day 0 in both groups R and F, with a significant relative abundance of 22.40% and 21.73%, respectively. Unknown members of *Mycoplasmataceae*, *Neisseriaceae* and *Oxalobacteriaceae* were also spotted.

The prediction of the bacterial profile at species level resulted in the identification of the following species: *Bifidobacterium animalis*, *B. bifidum*, *B. saeculare*, *Nocardioides albus*, *Bacillus coagulans*, *B. thermoamylovorans*, *Lacticaseibacillus casei*, *Lactiplantibacillus plantarum*, *Limosilactobacillus reuteri*, *Furfurilactobacillus rossiae*, *Leuconostoc citreum*, *L. lactis*, *Weisella cibaria*, *Staphylococcus aureus*, *Lactococcus raffinolactis*, *Streptococcus salivarius* subsp. *thermophilus*, *S. downei*, *Campylobacter jejuni*, *Pseudomonas oryzihabitans.*

**Fig. 6:**
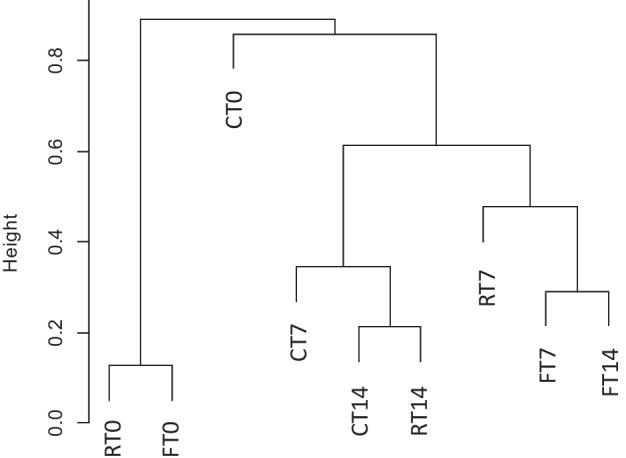
Hierarchical clustering analysis of the gut samples on the basis of microbiota structure at the end of the restriction/deprivation period (T0) and at the subsequent sampling times, (T7 and T14). C=control, R=restricted, F=fasted.

The results of hierarchical clustering analysis (Fig. 6) (supplementary information 1) demonstrated that on day 0, after the fasting and restricted period, group R and F (RT0 and FT0) were significantly distant from the control group (CT0). However, after the reintegration of the diet with 120% of the control ration, there was a restoration of the intestinal microbiota, which returned to a composition very similar to that of the control group.

## Discussion

Weight and length are useful parameters to describe fish growth. Their ratio in the condition factor also gives information on the state of fattening (low values indicate lean fish). In the present trial, the 3-week period of feed restriction, significantly affected the body weight. On the contrary, the condition factor was not affected by feed deprivation thus suggesting that the relation between fish mass and length, which is a structural feature of the fish, was maintained also during the fasting period probably also because of the initial size of the fish and the length of the restriction. Other authors, such as Boujard et al. (2000), have considered the consequences of fasting on trout with similar and significant effects on weight and growth. Similarly, alimentary restriction determines a smaller growth in terms of both weight and length in the sea bream (Bavčević et al. 2010). In this species, however, the effect on the fish mass was more marked than in trout, likely due to a different metabolic rate.

Regarding the VSI and HSI, the significantly low ratio in the F group at the end of the fasting period (T0), describes an important decrease not only in the body weight but also in viscera and liver weight. In fact, numerous ectotherm and endotherm animals have been reported to reduce the mass of their gastrointestinal tissues to decrease the energetic demands during starvation. Even liver may undergo a reduction in mass, albeit at lower rates, as its constituent nutrients are mobilized (see the review of McCue 2010). In fact, during the fasting period, the fish use the hepatic glycogen and lipid as an energy source to meet the metabolic requirements (Farbridge et al. 1992).

During the refeeding phase, the sharp increase in body weight of the fasted group registered on days 1 and 2 of refeeding was probably linked to the filling in of the digestive tract. In the following days, a partial compensatory growth was observed in R and F groups as the weight increases more in animals that had suffered feed restriction, but 2 weeks of refeeding and the increased amount of feed were not enough to restore the weight. Compensatory growth has already been reported in salmonids (Ali et al. 2003), however in some cases the compensation was only partial in mass growth (Miglav and Jobling 1989; Jobling et al. 1993) or did not involve the structural aspect, such as length (Álvarez and Nicieza 2005).

For the whole trial, feeding behaviour was strictly monitored. During the first 2 days of refeeding, fasted trout did not exhibit normal locomotory activity associated with feed intake, showing little interest in the pellets. As a consequence, feed distribution time for this group was longer than that of the C and R groups. Despite this initial difficulty, afterwards the fish were able to eat all the ration. In 7 days, the viscera/weight ratio reversed as a consequence of the reactivation of digestive activity. This is in agreement with Krogdahl and Bakke-McKellep (2005) who showed that the mass of the digestive tract of Atlantic salmon increased during the refeeding period as the consequence of the stimulation of feed on tissue regeneration. Moreover, at the end of the refeeding period, even the HSI reversed. It is likely that the stimulated metabolic activity in the liver and the availability of glucose from feed carbohydrates push the fish to restore the liver glycogen deposits, increasing liver weight (Farbridge et al. 1992; Bandeen and Leatherland 1997; Barcellos et al. 2010).

Glycaemia is well controlled in fish by the pancreatic hormones insulin and glucagon (Hazon and Balment 1998; McCue 2010; Navarro et al. 1992; Navarro and Gutierrez 1995; Weber et al. 2016). The former push the liver to accumulate glycogen and the latter stimulates the release of hepatic glucose when its plasma level decreases, as happens in fasting between meals. Cortisol is another important hormone to be considered in fasting and refeeding, but the evidence in fish is still contradictory (Pottinger et al. 2003; Barcellos et al. 2010; Dar et al. 2019). In the present trial, feed restrictions did not affect glycaemia which remained almost the same in all groups also during the refeeding period, thus confirming the ability of the adult rainbow trout to control plasma glucose concentration. Similar results were obtained by Barcellos et al. (2010) in adult jundiá and in sea bass and blackspot by Caruso et al. (2011), showing that different fish species subjected to fasting periods are able to maintain the homeostasis of glucose. Hepatic gluconeogenic processes (Moon et al. 1989; Furne´ et al. 2012) and lower glycolytic activity already reported in trout during starvation (Kirchner et al. 2005) could be the biochemical processes that regulate the homeostasis of glucose in this trial as well.

Treatment did not affect either plasma total protein or albumin, but the evaluation of these two parameters is not enough to understand if it depends on a reduction of the rate of the protein catabolism or on an increase in the protein mobilization. It is noteworthy that the constancy of the plasma protein and albumin concentration is of paramount importance in the osmoregulation and filtration processes that are viable in fish. As a consequence, animals are capable of well regulating the protein plasma concentration.

Circulating triglycerides were affected by both feed restriction and deprivation, suggesting that lipolysis was not able to compensate for low plasma level. After the first meal, during the refeeding period, all the treated fish were able to maintain values not different from the control group, showing that this parameter in rainbow trout is poorly controlled and depends on nutrient supply. Decreasing plasma triglycerides concentration has also been reported by Shimeno et al. (1997) in common carp *Cyprinus carpio* subjected to feed restriction or fasting. The authors measured a significant increase in free fatty acid that could explain the contribution of the lipid tissue in providing energetic molecules for cellular metabolism. Otherwise, in Asian sea bass, Norouzi et al. (2020) found an increase in plasma triglycerides after 4 and 8 days fasting due to the energetic demand of the fish.

On the contrary, plasma cholesterol was significantly affected by the feed quantity and the fasted group maintained a lower concentration than the fed fish. Fasted animals could reach the control values only after 14 days. Since cholesterol is an important element of the cellular membranes and the precursor of steroid hormones, the low level of the fasted group could be considered a disadvantageous condition, at least during the fasting period and the first 7 days of refeeding. A similar trend in values has been reported in trout and sturgeon subjected to long-term fasting and refeeding (Furne´ et al. 2012), and in common carp subjected to feed deprivation and starvation (Shimeno et al. 1997). Overall, it seems that plasma cholesterol is strictly related to the dietary intake rather than to the mobilization of the reserves (Furne´ et al. 2012).

When we want to evaluate the specific activity of the brush border enzymes, it is necessary to take into account the high individual variability, which is always present in the fish species and represents a critical point in the processing of data. This limit, however, did not prevent the study of the activities of the enzymes of the intestinal BBM over time and in different species (Villanueva et al. 1997; Harpaz and Uni 1999; Krogdahl et al. 1999, 2004; Krogdahl and Bakke-McKellep 2005; Tibaldi et al. 2006; Xu et al 2009; Vizcaino et al. 2014; Messina et al. 2019).

Studies performed on several fish species showed that the activity of the BBM enzymes is affected by changes in feed ingredients, starvation and feeding time (Al Hafedh 1999; Krogdahl et al 1999; Harpaz et al. 2005a, b; Tibaldi et al. 2006). The activity of the BBM enzymes is closely connected with the availability of partially digested saccharides and proteins such as disaccharides and di-tri peptides. At the same time, these enzymes are functionally expressed and active after short-term fasting in carnivorous fish like European sea bass (Messina et al. 2019) and after short and long-term fasting in Atlantic salmon (Krogdahl and Bakke-McKellep 2005). A decrease in the digestive enzyme activities has been observed after different periods of fasting though upon refeeding these enzyme activities were largely restored (Bélanger et al. 2002; Krogdahl and Bakke-McKellep 2005). Otherwise, fasting or refeeding did not affect the activity of digestive enzyme in *C. labrosus* (Pujante et al. 2015), thus suggesting species-specific differences.

In the present study, fish of the F group exhibited a depressed intestinal enzymatic activity at the end of the fasting period and during the first days of refeeding. It is interesting to notice the basal activity of the BBM enzymes in the F group as the result of the physiological activity of the intestinal epithelial cells that provides information on the adaptability of rainbow trout to long-term fasting periods. The decreased activity should be considered as a consequence not only of the lack of the stimuli from the meal but also of the loss of intestinal tissue described by the significant low VSI value. In the R group, the restriction of 30% of the C ration did not have any significant effect (T0), thus suggesting that the presence of the *digesta*, even if in a reduced quantity, stimulates the activity of the BBM enzymes.

During the refeeding period, the restoration of enzymatic activity in R and F group presents different patterns, with the values of the R group almost always similar to the control group, suggesting that the feeding history, in term of diet quantity, is important in determining the activity during the refeeding period.

Overall, during the refeeding period, the activity of the BBM enzymes in the three tracts of the gut, showed considerable variability and eventually converged on similar values after 14 days. This high variability occurred in particular after a few days of refeeding and could be due to the first attempts of response by the fish to cope with the *digesta* in the intestine. In fact, MALT, SI and γ-GT are enzymes stimulated by the presence of their substrate, while the IAP is considered a marker of the maturity of enterocytes (Gisbert et al. 2018). Over time, with the resumption of feeding and the consequent constant stimulus of the intestinal content, the specific activity of the enzymes reached similar values in the different groups.

Intestinal microbiota plays the crucial roles of maintaining the regular functions of the host’s immune system and organs as well as that of contributing to the nutrition of the host (Yang et al. 2017; Hooper et al. 2012; Einar et al. 2014; Hoseinifar et al. 2015, 2016; Tang et al. 2017). Fish intestinal microbiota actively participate in digestion and this impacts the actual growth, reproductive capacity, population dynamics and above all the state of health and vulnerability to diseases (Ghanbari et al. 2015; Dulski et al. 2020). Among the different factors which can affect microbiota homeostasis, diet and environmental characteristics are the most effective and, as demonstrated by Liu et al. (2020) and Michl et al. (2019), starvation and refeeding can cause changes in growth, non-specific immunity and microbiota balance.

The characterization of the microbial community of the rainbow trout intestine in response to feed restriction or fasting by 16SRNA V3 region sequencing was in accordance with previous results, demonstrating that Tenericutes, Firmicutes, Proteobacteria and Spirochete were the dominant core (Michl et al. 2019; Parshukov et al. 2019; Wong et al. 2013; Lyons et al. 2017a, 2017b). However, in this study, Actinobacteria were found as the most represented phylum (relative abundance, %), along with Proteobacteria and Firmicutes, according to Rimoldi et al. (2018) and Betiku et al. (2018). Tenericutes, a phylum associated to unhealthy hybrid grouper by Liu et al. (2020), was not observed in the control group subjected to continuous feeding. Similarly, Parshukov et al. (2019) demonstrated a higher relative abundance of Actinobacteria in unhealthy rainbow trout than in the healthy control group. On the other hand, other authors observed that Actinobacteria were an essential part of the core microbiota, but there is still a lack of insights into their function and their real impact on animal welfare (Wang et al. 2021; Yang et al. 2017; Michl et al. 2019). Bacteroidetes were not detected, although they have already been associated with fish fed with a plant-based diet (Michl et al. 2019). In the control group Actinobacteria, Firmicutes and Proteobacteria remained predominant during the entire monitored period, although at 7 and 14 days, there was an increase in Proteobacteria and a concomitant decrease of Actinobacteria.

A different situation was found at the end of the restriction and fasting period in groups R and F. Both the treatments had a very similar effect on the core microbiota, resulting in an increment of the biodiversity and a significant reduction of the Actinobacteria group, which was replaced by Bacteroidetes, and other unknown microbes of Bacteria kingdom (16.92% and 25.54% for R and F, respectively). In humans, *Bacteriodes* spp. are able to complete the digestion of a variety of vegetable dietary components, such as starch and cellulose (Hooper et al. 2002). In this way, they become more competitive than other microorganisms and provide the host with metabolizable energy during the restriction/starvation period.

A higher relative abundance of Firmicutes was also observed as well as Fusobacteria and Tenericutes, confirming that a reduction of the ration and fasting result in a substantial impact on dysregulation of gut microbiota homeostasis, allowing for the development of other commensal or pathogenic bacteria (Liu et al. 2020; Michl et al. 2019). Moreover, gut perturbation can be more evident if the feed restriction or fasting are combined with a stressed immune system (Perry et al. 2020). On this basis, looking at the family and genera level of microorganisms, dysbiosis due to nutritional stress led to a drastic decrease in Bifidobacteriaceae, with the genus *Bifidobacterium* falling below 1% of relative abundance. Bifidobacteria are widespread in the intestinal microflora of both invertebrate and vertebrate animals, demonstrating their involvement in the maintenance of a healthy fish state, together with *Lactobacillus* spp. (Wang et al. 2021; Parshukov et al. 2019; Vlková et al. 2012; Rimoldi et al. 2018). Michl et al. (2019), in addition, demonstrated that lactic acid bacteria (LAB) and Bifidobacteria are influenced by the type of diet, whether based on fish protein or vegetable protein. The positive effect of LAB and Bifidobacteria on fish health could be understood from the knowledge on humans, in which they provide an additional source of vitamins, affect the immune system and can inhibit the growth of different pathogens. In fact, from the data obtained by Askarian et al. (2012), it emerged that the decrease in the genus *Bifidobacterium* is correlated to an increase in potential pathogenic microorganisms in fish subjected to fasting and restricted diet*. Neisseriaceae*, *Micoplasmataceae*, *Staphylococcus* spp. and particularly *Pseudomonas* spp. increased significantly after the stressful nutritional regimen in both R and F groups. The latter are commonly recognized as plant, animal, humans and fish pathogens and opportunistic pathogens (Wakabayashi et al. 1996; Kusuda and Toyoshima 1976). As fish pathogens, they are responsible for severe economic losses in the aquaculture industry. Among the various species, *P. putida* and *P. fluorescens* are recognized as the major rainbow trout pathogens, but also species as *P. aeruginosa*, *P. anguilliseptica*, *P. tructae* and *P. plecoglossicida* were reported as responsible for mortality in rainbow trout (Berthe et al. 1995; Altinok et al. 2006; Sakai et al. 1989; Oh et al. 2019a, 2019b; Duman et al. 2019). Lachnospiraceae, in accordance with the findings of Liu et al. (2020), were only found in R and F groups. The role of Lachnospiraceae is still controversial and the majority of the published studies are related to humans. Due to their high capability to produce short chain fatty acids, they are often associated with a beneficial impact on the host. On the other hand, there are many pathologies (metabolic, liver, kidney, and inflammatory bowel diseases) for which the substantial changes in Lachnospiraceae composition seemed to be more responsible than other co-factors (Vacca et al. 2020).

It is worth noting that the dysbiosis caused by the restricted diet and fasting allowed a significant increase in the genus *Helicobacter*, which reached about 20% of relative abundance. Our finding is in accordance with other authors, who observed in different animals and humans a similar increment in the cases of disturbing events in the balance of the gut microbiota (Menard and Smet 2019). To our knowledge, this is the first study that has demonstrated that even in fish many pathogenic species of this genus, including *Helicobacter pylori* as the main pathogenic, are favored in case of alteration of the gut microbiota. Previously, this had been reported for dogs, pandas, piglets, macaques, chickens, poultry, and mice, for which different *Helicobacter*, species (*H. pilori*, *H. winghamenis*, *H. macacae*, *H. brantae*, *H. hepaticus*, *H. suis*, *H. felis*, *H. cinaedi*) were responsible for several diseases, such as gastric cancer, vertebral osteomyelitis, proliferative canine tumors and idiopathic chronic diarrhea, (Guo et al. 2018; Herstad et al. 2018; Adhikari et al. 2019; Wang et al. 2018; Laing et al. 2018; Kollarcikova et al. 2019).

As expected, therefore, also in our study, the resumption of feeding at full capacity of 120% of C group ration, effectively restored the balance in the intestinal microbiota homeostasis. Furthermore, data showed that the microbial community in the trout intestine can be reestablished at the 7^th^ day in response to refeeding, as can be seen from the results of the hierarchical cluster analysis.

The fish gut microbiota could affect also the digestive performance, by producing enzymes that can help the digestive processes of the host (Xu et al. 2009) or affecting the enzymatic activity of the host. Data on the activity of BBM enzymes in relation to the gut microbiome in fish are very scarce and refer to the IAP study (Bates et al. 2006, 2007). In a recent work, Lallès (2020) underlined that an increment in the intestinal concentration of *E. coli* showed a slight negative correlation with increased IAP activity in jiang carp. Other considered microbial species, such as *Aeromonas hydrophila* and *Lactobacilli* did not show the same effect. The metagenomic approach that was used in the present study, did not allow to take such conclusion and to understand if there is a direct impact on the enzymatic activity by the presence of specific microbial concentrations. This is due to the fact that NGS allows to evaluate the microbial ecology in terms of relative abundance, but without knowledge on absolute quantification of the present microbial species, any hypothesis of the impact of the different microbial species on the enzymatic activity of the fish would be too speculative.

In conclusion, data showed that the main reaction to refeeding from day 1 is the recovery of the functionality of the digestive system rather than synthesize new muscle tissue, which did not seem to be a priority in the considered time interval. Moreover, after seven days there is the rebalancing of the intestinal microbiota. It has to be highlighted that this is the first time that pathogenic bacteria were observed emerging due to the dysbiosis determined after the restriction period in rainbow trout. This aspect is of great concern for trout welfare. These findings could provide insights for intestinal microbiota manipulation in trout, for potential application in aquaculture practice.

## Supplementary Information

Below is the link to the electronic supplementary material.

Supplementary file1 (DOC 34 KB)

## Data Availability

The datasets generated and analysed during the current study are available from the corresponding author on reasonable request.
