# Supplementary material for: Effect of feed restriction and refeeding on body condition, digestive functionality and intestinal microbiota in rainbow trout (Oncorhynchus mykiss)
Source: Fish Physiol Biochem. 2023 Jan 21;49(1):169–89. doi: 10.1007/s10695-023-01170-z (PMC9935662; doi:10.1007/s10695-023-01170-z)
Supplement: Supplementary file 1 — Supplementary file1 (DOC 34 KB) [file 10695_2023_1170_MOESM1_ESM.doc]

Table S1. Ingredient composition of the rainbow trout diet*

|  | g/100g  *as fed* |
| --- | --- |
| Fish meal | 25.0 |
| Corn gluten meal | 20.0 |
| Soybean meal | 10.0 |
| Fish Oil | 8.5 |
| Rapeseed oil | 8.0 |
| Wheat | 8.0 |
| Rapeseed meal | 5.0 |
| Guar meal | 5.0 |
| Wheat gluten | 3.5 |
| Sunflower meal | 3.5 |
| Soy Protein Concentrate | 3.0 |
| Vitamin/mineral mix | 0.5 |

*Courtesy provided by the manufacturer Skretting Italia SpA
